# Association of thalamic hyperactivity with treatment-resistant depression and poor response in early treatment for major depression: a resting-state fMRI study using fractional amplitude of low-frequency fluctuations

**DOI:** 10.1038/tp.2016.18

**Published:** 2016-03-08

**Authors:** T Yamamura, Y Okamoto, G Okada, Y Takaishi, M Takamura, A Mantani, A Kurata, Y Otagaki, H Yamashita, S Yamawaki

**Affiliations:** 1 Department of Psychiatry and Neurosciences, Graduate School of Biomedical and Health Sciences, Hiroshima University, Hiroshima, Japan; 2 Mantani Mental Clinic, Hiroshima, Japan; 3 Department of Psychiatry, Hiroshima City Hiroshima Citizens Hospital, Hiroshima, Japan; 4 Kyobashi Shinryo Clinic, Hiroshima, Japan

## Abstract

Despite novel antidepressant development, 10–30% of patients with major depressive disorder (MDD) have antidepressant treatment-resistant depression (TRD). Although new therapies are needed, lack of knowledge regarding the neural mechanisms underlying TRD hinders development of new therapeutic options. We aimed to identify brain regions in which spontaneous neural activity is not only altered in TRD but also associated with early treatment resistance in MDD. Sixteen patients with TRD, 16 patients with early-phase non-TRD and 26 healthy control (HC) subjects underwent resting-state functional magnetic resonance imaging. To identify brain region differences in spontaneous neural activity between patients with and without TRD, we assessed fractional amplitude of low-frequency fluctuations (fALFF). We also calculated correlations between the percent change in the Hamilton Rating Scale for Depression (HRSD_17_) scores and fALFF values in brain regions with differing activity for patients with and without TRD. Patients with TRD had increased right-thalamic fALFF values compared with patients without TRD. The percent change in HRSD_17_ scores negatively correlated with fALFF values in patients with non-TRD. In addition, patients with TRD showed increased fALFF values in the right inferior frontal gyrus (IFG), inferior parietal lobule (IPL) and vermis, compared with patients with non-TRD and HC subjects. Our results show that spontaneous activity in the right thalamus correlates with antidepressant treatment response. We also demonstrate that spontaneous activity in the right IFG, IPL and vermis may be specifically implicated in the neural pathophysiology of TRD.

## Introduction

Major depressive disorder (MDD) is a common psychiatric illness, characterized by persistent depressed mood, anxiety, dysphoria and alterations in psychomotor functions, motivation, social behavior and sleeping patterns.^1^ Although many people with depression have been successfully treated using several classes of antidepressants, approximately 10–30% of patients do not respond to standard antidepressant treatments.^2, 3^ Treatment-resistant depression (TRD) is defined as a lack of clinically meaningful improvement following the use of least two different antidepressants prescribed at adequate dosages and durations with confirmation of adherence to treatment protocols in a regulatory setting.^4^ A better understanding of the biological pathogenesis of TRD is required to rapidly detect patients who are likely to develop treatment resistance, and to develop more effective therapeutics for these patients.^5^

One of the possible methods used to reveal the biological mechanisms underlying TRD is resting-state functional magnetic resonance imaging (fMRI). fMRI can measure task-independent and task-specific neural function, and resting-state fMRI can assess task-independent neural function in particular.^6^ The most popular approaches are region-of-interest functional connectivity (FC)^7^ and independent component analysis.^8^ In both methods, brain regions are compared to determine whether there are synchronized changes in activation over time.^8^ If regions exhibit temporally common behavior, they are thought to be functionally connected, even if they are not structurally connected.^8, 9^ Although infrequently used, another resting-state fMRI method assesses the amplitude of low-frequency fluctuations (ALFF).^10^ Because ALFF is higher in gray matter than in white matter,^9^ and observed neural activity in the visual cortex is high due to low-frequency fluctuations assessed using the power spectrum method,^11^ it is thought to reflect spontaneous neural activity.^12, 13^ Although these methods assess task-independent neural function/activity, previous studies have revealed associations between resting-state FC/ALFF and task-evoked neural activity.^14^ In addition, the functions of the various regions intrinsically connected during the resting state have been suggested, such as pertaining to emotion, memory, action and vision.^15^ In summary, as resting-state fMRI approaches might reveal the neural function/activity associated with cognitive abilities and mood reactivity, these approaches may be appropriate for investigating the neural substrates of TRD.

Several studies have identified some of the neural substrates underlying TRD by using the aforementioned resting-state fMRI. Using independent component analysis, patients with depression showed increased network functional connectivity in the subgenual cingulate and the thalamus,^8^ and decreased FC was found in the cerebellum, precuneus and inferior parietal lobule (IPL) in patients with TRD compared with patients who had treatment-sensitive depression.^16^ A regional homogeneity approach, based on Kendall's coefficient of concordance (KCC-ReHo),^17^ revealed that patients with TRD had increased KCC-ReHo values in the left superior temporal gyrus and cerebellar posterior lobe (tuber), anterior lobe (culmen) and right tonsil.^18^ In contrast, patients with TRD had decreased KCC-ReHo values in the left insula, superior temporal gyrus, inferior frontal gyrus (IFG), lingual gyrus and cerebellum anterior lobe (culmen).^18^ Assessment of coherence-based regional homogeneity^19^ revealed that patients with TRD showed increased coherence-based regional homogeneity values in the left fusiform gyrus and left cerebellum compared with patients with treatment-sensitive depression, and decreased values in the bilateral superior frontal gyrus compared with healthy control subjects.^20^ Another study reported a positive correlation between higher fractional amplitude of low frequency fluctuations (fALFF)^21^ values and depressive symptoms in treatment naïve patients,^22^ and ALFF^13^ values in the posterior lobes of the cerebellum and the default mode circuit (anterior cingulate cortex and medial frontal gyrus) and lower ALFF values in the visual recognition circuit (cuneus and lingual, middle occipital and middle temporal gyri) in patients with TRD compared with healthy control (HC) subjects and patients with treatment-sensitive depression.^23^

In contrast, neuroimaging studies have also identified brain regions that are associated with responsiveness to pharmacotherapy in patients with MDD. For instance, in a resting-state fMRI study, disrupted FC between the frontal lobes and thalamus was associated with treatment resistance in patients with MDD.^24^ Thalamic metabolism in depressive patients decreased along with their remission^25^ and resting-state FC between the medial thalamus and dorsal anterior cingulate normalized following treatment with sertraline in depressed patients.^26^ Another study demonstrated that treatment resistance was associated with increased KCC-ReHo values in the right insula, bilateral anterior cingulate cortex and bilateral medial frontal gyrus.^27^ Treatment resistance was also associated with decreased KCC-ReHo values in the left lateral frontal gyrus, bilateral IPL and left superior parietal lobule.^27^ A study utilizing 2-[^18^F]fluoro-2-deoxy-d-glucose positron emission tomography revealed that in the rostral cingulate gyrus, drug treatment in nonresponsive patients with MDD decreased glucose metabolism, whereas this parameter increased in treatment-responsive patients.^28^ A voxel-based morphometry study showed that differences in the frontal, temporal, parietal, occipital and subcortical regions are strong predictors of treatment responsiveness before antidepressant use,^29^ and associated with the time between initiation of treatment and recovery.^30^ A separate study demonstrated that stronger task-related activations in the anterior mid-cingulate in addition to the pregenual and subgenual anterior cingulate cortices were associated with a more rapid decrease in depressive symptoms.^30^ Moreover, hippocampal activation in response to images depicting faces with happy emotions correlated with treatment responsiveness after 8 weeks of treatment.^31^ In addition, subgenual cingulate and parahippocampal region activation in response to images of sad faces predicted a strong response to antidepressant treatment.^32^

The results of these studies suggest that patients with TRD have different resting-state spontaneous neural activity compared with patients with treatment-sensitive depression. Moreover, these studies indicate that in some brain regions, spontaneous neural activity, neural responses to facial emotions and gray matter volumes predict antidepressant treatment responsiveness. We therefore hypothesized that different spontaneous regional neural activity in patients with TRD and non-TRD would be associated with antidepressant treatment responsiveness in the early phase of treatment. The purpose of this study was to not only determine brain regions in which spontaneous neural activity was altered in patients with TRD, but also to identify brain areas associated with antidepressant responsiveness in patients with non-TRD. In addition, we investigated the brain regions in which spontaneous neural activity differed between patients with TRD, patients with non-TRD and HC subjects.

## Materials and methods

### Participants

Healthy volunteers were recruited in the control group. Volunteers were included if they were aged between 25 and 75 years. Subjects were excluded from the control group on the basis of the following criteria: (a) history of psychiatric illness, or current psychiatric symptoms, as determined by the Mini International Neuropsychiatric Interview,^33^ (b) diagnosis of neurological illness, (c) left-handedness, as defined by a score less than 0 on the Edinburgh handedness test.^34^

The non-TRD group included patients who were either untreated or treated with a single antidepressant at an insufficient dose and duration and were recruited from the Hiroshima University and regional hospitals according to the following inclusion criteria: (a) age between 25 and 75 years, (b) outpatient status, (c) presentation of depressive symptoms, as determined by a score on the Hamilton Rating Scale for Depression (HRSD_17_)^35^ of 8 or more,^36^ (d) diagnosis of non-psychotic MDD and current depressive episode, as determined by an experienced psychiatrist according to the Diagnostic and Statistical Manual of Mental Disorders, Fourth Edition, Text Revision (DSM-IV-TR).^1^ The exclusion criteria for this group were as follows: (a) diagnosis of neurological illness, current or previous psychotic disorder, current high risk of suicide, current or previous substance abuse and serious somatic disease as determined by the Mini International Neuropsychiatric Interview^33^ conducted by trained valuators, (b) left-handedness, which was defined as a score less than 0 on the Edinburgh handedness test,^34^ (c) current pregnancy or nursing, (d) sufficient treatment and duration with one antidepressant to treat the current episode of depression, (e) use of two separate antidepressants for the current episode of depression, (f) use of mood stabilizers, antipsychotics or central nervous system stimulants, (g) treatment with electroconvulsive therapy within the past 3 months.

Patients who were experiencing TRD according to the criteria established by European Medicines Agency guidelines^4^ (treatment-resistance level of at least stage 2, according to the Thase and Rush^37^ definition) were recruited using inclusion criteria similar to those for patients with non-TRD. Patients with TRD were excluded using only criteria (a), (b) and (c) from the exclusion criteria used for patients with non-TRD. All the aforementioned inclusion and exclusion criteria were established before recruiting.

Because we used a liberal threshold of *P*<0.05, about 12 subjects were required to achieve 80% power at the single voxel level for a typical activation study.^38^ Consequently, we aimed to recruit more than 12 participants in each group.

### Ethical approval and consent

This study protocol was approved by the Ethics Committee of the Hiroshima University Graduate School of Biomedical and Health Sciences. Written informed consent was obtained from all the participants.

### Clinical assessments

After receiving informed consent, we evaluated the severity of depressive symptoms for patients with TRD and non-TRD. To evaluate depressive symptoms, HRSD_17_^35^ was used. Verbal intelligence was assessed by the Japanese Adult Reading Test.^39^ Patients with TRD were assessed for treatment resistance by using the Maudsley staging method (MSM).^40^ MSM assesses treatment resistance according to five factors: duration of presenting episode, severity of depression, antidepressant treatment failure, augmentation therapy use and electroconvulsive therapy use. In addition, patients with non-TRD were assessed by the HRSD_17_ following 6 weeks of selective serotonin reuptake inhibitor treatment to evaluate clinical improvement. Improvement was determined according to the percent change in HRSD_17_ scores, as calculated by the following formula: percent change=[{(HRSD_17_ score before medication)−(HRSD_17_ score after 6 weeks of treatment)}/(HRSD_17_ score before medication)] × 100.

### Magnetic resonance imaging

After clinical assessment, all the participants underwent a 5-min, whole-brain resting state fMRI and three-dimensional anatomical scans at the Kajikawa Hospital for evaluation of the spontaneous resting-state neural activity. MRI acquisition was performed using a Magnetom Spectra 3T scanner (Siemens, Tokyo, Japan). A quadrature birdcage head coil was used to minimize the head movement. Before scanning, all the participants were instructed to remain motionless, keep their eyes closed, not think of anything in particular and refrain from sleeping to maximally reduce physiological noise in the fMRI data. A total of 112 volumes were recorded over 5 min using a gradient-echo T2*-weighted echo planar imaging sequence (TR/TE=2700/31 ms, 38 slices, 64 × 64 matrix, 90° flip angle, 19.2 cm field of view, 3 mm slice thickness and no gap). Anatomical images were recorded over 8 min using a T1-weighted gradient-echo pulse sequence (TR/TE=1900/2.38 ms, 224 slices, 320 × 320 matrix, 10° flip angle, 24 cm field of view, 0.8 mm slice thickness and 0.2 mm gap).

### Imaging preprocessing

Data were preprocessed using the Data Processing Assistant for Resting-State fMRI^41^ software. The first 10 images for each session were discarded to allow for a steady state in longitudinal magnetization and participant habituation to the scanning environment. The remaining 102 images were realigned to the initial image to correct for movement. Subjects who had excessive head motion (>1.5 mm translation or 1.5° rotation) during the scan were excluded from further analysis. The functional images were then preprocessed, including slice timing correction and head motion correction, using a least squares approach with a six-parameter spatial transformation, and normalized to the Montreal Neurological Institute template (with a resampling voxel size of=3 × 3 × 3 mm). MRI images were then smoothed with an isotropic Gaussian kernel (with a full width at half maximum of 8 mm) and linear image trends were removed.

### fALFF calculation

To determine spontaneous neural activity, we calculated the fALFF using the Data Processing Assistant for Resting-State fMRI,^41^ as defined previously.^21^ Although reporting both ALFF and fALFF values is recommended,^10^ we used only the fALFF method for the following three reasons. First, fALFF minimizes artifacts due to body motion, respiration and cardiac noise;^42^ it has been noted that low-frequency oscillation approaches are vulnerable to these effects.^42^ Second, compared with ALFF, fALFF has moderate intra- and inter-session test–retest reliability and a high sensitivity for detecting spontaneous neural activity in gray matter.^10^ Third, fALFF is more sensitive to spontaneous neural activity in the default mode network (DMN) than ALFF. Of relevance to this is a recent meta-analysis that reported hyper-connectivity in the resting-state in MDD.^43^ This technique has also been successfully used to detect altered spontaneous neural activity in patients with MDD.^44^ In many resting-state fMRI studies, preprocessed data is band-pass filtered in the low-frequency range (0.01–0.08 Hz) to reduce ultra-low-frequency drift and high-frequency respiratory and cardiac noise.^9, 45^ In this study, fMRI signal time series data for each voxel were transformed to the frequency domain, and the power spectrum of the full band (0–0.25 Hz) was obtained. This approach was used because fALFF values were defined as the ratio of the power of each frequency at the low-frequency range (0.01–0.08 Hz) to that of the entire range (0–0.25 Hz). The square root was calculated for each power spectrum frequency, and the amplitude sum across 0.01–0.08 Hz was divided by that of the entire frequency range. We determined the aforementioned parameters for fALFF preprocessing and calculation according to previous research using this approach.^44^ Although we do not report ALFF analysis here, we do so in the Supplementary Materials (see Supplementary Tables 2 and 3). In the supplementary ALFF analysis, the square root of the power spectrum between 0.01 and 0.08 Hz was calculated, as in the fALFF analysis, but was not divided by the sum over the entire frequency range.

### Data analysis

For demographic and clinical variables, statistical analysis was carried out using the Statistical Package for the Social Sciences software, version 20 (IBM, Tokyo, Japan). We conducted the Shapiro–Wilk test and Levene's test to assess normality of distributions and equity of variance among variables. Subsequently, we conducted analyses of variance to test for differences for these variables among groups.

After preprocessing imaging data, we performed group analyses. To test our hypothesis that patients in the TRD, non-TRD and HC groups have different spontaneous neural activities, we conducted one-way ANOVA for fALFF at each voxel using Statistical Parametric Mapping 8 software (http://www.fil.ion.ucl.ac.uk/spm), with age and sex as covariates. We then conducted the two-tailed, two-sample *t*-tests (TRD compared with non-TRD, TRD compared with HC and non-TRD compared with HC groups) for differences in fALFF values undetected by ANOVA. Because fMRI data in each group may not be equally distributed, even after removing the effect of artifacts by the above procedure, we conducted statistical analysis for fALFF under the assumption of unequal distributions. In addition, although the mean ages of three groups were not statistically different in this study, as a previous study showed an association between age and fALFF values,^46^ we included age as a covariate in the statistical analysis. Then, we set an uncorrected significance level of *P*<0.005 and a cluster size of *k*≥10, accounting for type I and type II errors.^47^ Brain regions with statistically significant differences in fALFF values were labeled using Anatomical Automatic Labeling software.^48^ To determine the brain regions associated with clinical improvement and treatment resistance, first we conducted partial correlation analysis between fALFF and percent change in HRSD_17_ scores in patients in the non-TRD group, using R^49^ software ver. 3.1.1. and ppcor, removing the duration of the current episode (month). Second, to determine the brain regions linearly associated with the spontaneous neural activity and treatment resistance, we conducted partial correlation analysis between fALFF and MSM scores in the TRD group, using R^49^ software and ppcor. Third, to determine the brain regions nonlinearly associated with fALFF and MSM scores, we calculated the maximal information coefficient^50^ using R^49^ software and minerva (http://mpba.fbk.eu/cmine). Mean fALFF values were extracted for the brain regions identified by two-sample *t*-tests (TRD compared with non-TRD groups) using the Mars Bar toolbox,^51^ because these regions include the effect of medication, duration of illness and treatment nonresponsiveness. We obtained the *P-*value for maximal information coefficient for a sample size of 20 from an online table (http://www.exploredata.net/Downloads/P-Value-Tables). Statistical significance for two-tailed no correlation analysis was set at *P*<0.05.

## Results

### Demographic and clinical characteristics

Data from 26 healthy volunteers, 16 patients with non-TRD and 16 patients with TRD (resistance severity according to Thase and Rush:^37^ Stage 2, *n*=7; Stage 3, *n*=8; Stage 5, *n*=1) were analyzed. Age, sex, age of onset and intelligence quotient were not statistically different among the three groups. Duration of current episode was longer in patients with TRD than non-TRD (Mann–Whitney's *U*=12.5, *P*<0.01). Both groups of patients showed moderate severity of depressive symptoms and depression severity (according to the HRSD_17_) was not significantly different between the patients with TRD and non-TRD before selective serotonin reuptake inhibitor treatment. Depressive symptoms among patients with non-TRD significantly decreased after 6 weeks of medication (*t*_(15)_=3.34, *P*<0.01). Demographic and clinical characteristics of the participants, including data for patients with non-TRD after 6 weeks selective serotonin reuptake inhibitor treatment, are shown in Table 1.

Treatment-resistance characteristics in patients with TRD, as assessed by the MSM, are shown in Table 2. Most of the patients were chronically depressed (87.5%), and showed at least mild symptoms (93.8%). None of the patients had comorbid psychotic symptoms. Among the patients, 75.0% had received at least three antidepressants and 68.8% had received augmentative medications. One patient had received electroconvulsive therapy. According to the MSM, 31.2% (*n*=5) had mild, 56.3% (*n*=9) had moderate and 12.5% (*n*=2) had severe treatment resistance. Supplementary Table 1 shows a summary of medication use at the time of MRI acquisition among patients with MDD.

### Group differences in fALFF values

**Figure 1:**
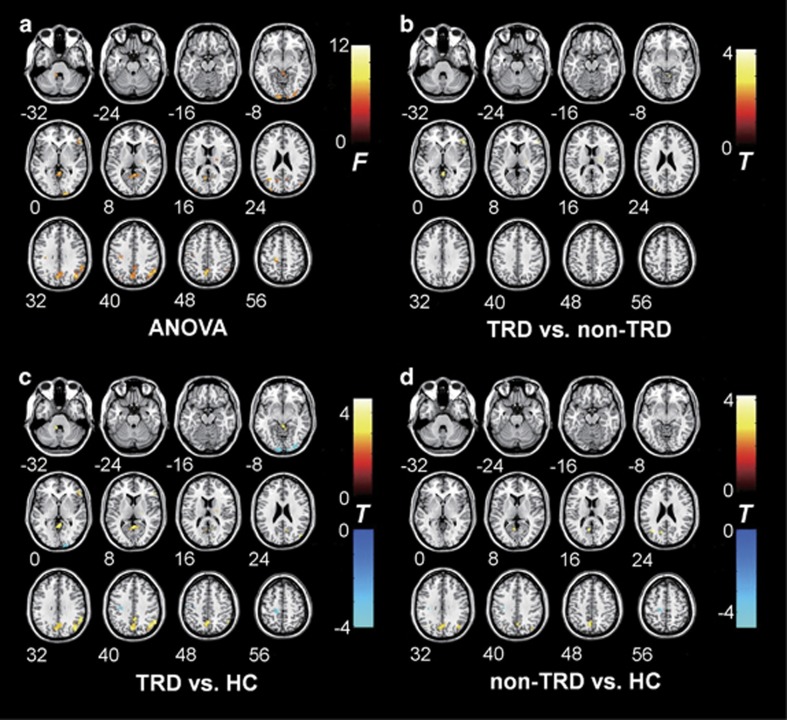
Brain regions showing differential fALFF values among groups. Statistical *F*- and *t*-maps show the results of the one-way analysis of variance (ANOVA) and two-sample *t*-tests for each fALFF value. The significance level was set at *P*_uncorrected_<0.005, with a cluster size of *k*≥10. (**a**) fALFF value differences determined by one-way ANOVA. (**b**–**d**) Two-sample *t*-test results showed significant group differences undetected by one-way ANOVA. Analyses between groups are shown as follows: TRD vs non-TRD (**b**), TRD vs HC (**c**) and non-TRD vs HC (**d**). fALFF, fractional amplitude of low-frequency fluctuation; HC, healthy control; TRD, treatment-resistant depression. Color bar indicates *F*- or *t*-values.

Compared with patients in the non-TRD group, patients with TRD showed increased fALFF values in the right IFG, right middle occipital gyrus, right thalamus, right IPL and vermis. Compared with those in the non-TRD and HC groups, patients with TRD showed increased spontaneous neural activation in the right IFG, right thalamus, right IPL (supramarginal gyrus) and vermis. In addition, compared with HC subjects, patients with MDD (those in the TRD and non-TRD groups) had common fALFF value increases in the precuneus and angular gyrus, and common decreases in the pre- and postcentral gyri. Moreover, patients with TRD showed decreased fALFF values in the bilateral calcarine cortex and left para- and pre-central gyri compared with HC subjects. Patients with non-TRD also had decreased fALFF values in left pre- and postcentral gyri compared with HC subjects. Figure 1 shows statistical *F*-value and *t*-value maps of one-way ANOVA and two-sample *t*-tests for each fALFF value (*P*_uncorrected_<0.005, cluster size: *k*≥10). Table 3 shows the two-sample *t*-test results for fALFF value comparisons (*P*_uncorrected_<0.005, cluster size: *k*≥10).

### Correlation between fALFF values and percent change in HRSD_17_ scores in the non-TRD group

**Figure 2:**
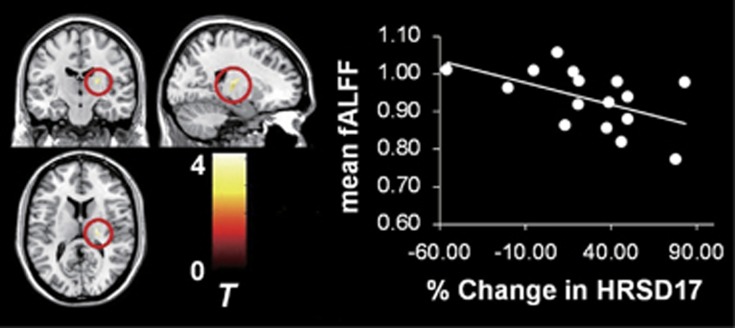
Correlation between percent change in HRSD_17_ scores and thalamic fALFF values for the non-TRD group. Statistical *t*-map indicates two-sample *t*-test results (TRD compared with non-TRD) for the right thalamus. The significance level was set at *P*_uncorrected_<0.005, with a cluster size of *k*≥10. Scatter plot depicts the relationship between percent change in HRSD_17_ scores and fALFF values for the right thalamus in patients with non-TRD (*n*=16). fALFF, fractional amplitude of low-frequency fluctuation; HRSD, Hamilton Rating Scale for Depression; TRD, treatment-resistant depression.

In patients with non-TRD, only the right thalamus showed negative correlation between mean fALFF values and percent change in HRSD_17_ scores (*r*=−0.519, *t*_(13)_=−2.187, *P*=0.029; Figure 2) after removing the effect of the duration of the current episode. There was no significant partial correlation between mean fALFF in the detected regions and percent change in HRSD_17_ (inferior frontal gyrus: *r*=0.333, *t*_(13)_=1.277, *P*=0.224; middle occipital gyrus: *r*=0.376, *t*_(13)_=1.463, *P*=0.143; supramarginal gyrus: *r*=−0.043, *t*_(13)_=−0.155, *P*=0.876; vermis: *r*=−0.087, *t*_(13)_=−0.315, *P*=0.753).

### Linear and nonlinear correlations between fALFF values and MSM scores in the TRD group

No significant linear correlations were detected (inferior frontal gyrus: *r*=0.083, *t*_(13)_=0.299, *P*=0.765; middle occipital gyrus: *r*=−0.275, *t*_(13)_=−1.030, *P*=0.303; thalamus: *r*=0.201, *t*_(13)_=0.741, *P*=0.459; supramarginal gyrus: *r*=−0.332, *t*_(13)_=−1.269, *P*=0.204; vermis: *r*=0.143, *t*_(13)_=0.521, *P*=0.602). In addition, nor were there any significant nonlinear correlations (inferior frontal gyrus: maximal information coefficient=0.138, middle occipital gyrus=0.138, thalamus=0.219, supramarginal gyrus=0.311, vermis=0.311; all *P*-values ≥0.05).

## Discussion

To the best of our knowledge, this is the first study to identify the brain regions in which spontaneous neural activity is associated with antidepressant treatment resistance in major depression. We combined both cross-sectional comparisons of patients with TRD and non-TRD (although these were early treatment-phase tests, and potentially include patients with TRD) with a prospective follow-up of the non-TRD group. Through this approach, we provided novel evidence that higher spontaneous resting-state neural activity in the thalamus might be a marker for treatment resistance. In the right thalamus, patients with TRD showed increased spontaneous neural activity compared with those with non-TRD. In addition, patients with non-TRD who had higher spontaneous neural activity showed lower clinical improvement on the HRSD_17_.

Recently, evidence of an association between the thalamus and MDD has been reported. For instance, in the context of resting-state neural activity, previous studies have suggested an association between spontaneous neural activity in the thalamus and TRD.^22, 25, 52, 53, 54^ Patients with MDD have been reported to exhibit greater neuronal density in the thalamus,^52^ greater regional cerebral blood flow therein^53^ and right-thalamic fALFF positively correlates with depressive symptoms.^22^ Thalamic metabolism decreases along with remisson,^25^ but increases after tryptophan depletion.^54^ Thus, although previous studies have suggested a possible association between thalamic hyper-spontaneous activity and TRD, the study reported here is the first to demonstrate this relationship directly using fALFF. Furthermore, in the context of resting-state FC, patients with TRD show decreased FC within the thalamo–cortical circuit^24^ and increased FC within the thalamus-subgenual ACC,^8^ which normalize after selective serotonin reuptake inhibitor treatment.^26^ These previous reports suggest alterations in functional connectivity and spontaneous neural activity in the thalamus in TRD in the resting state. The thalamus has a key role in the basal ganglia–thalamo–cortical circuits.^55^ In the context of mood disorders, three major prefrontal–striatal–pallidal–thalamic networks have been recognized.^56^ The first originates in the orbital/ventrolateral prefrontal cortex and appears to manage the emotional salience of external stimuli, as this network has strong connections to a variety of sensory brain regions. The second originates in the medial prefrontal cortex and appears to modulate internal mood states, given its connections to hypothalamic and consequently autonomic areas that presumably underlie emotions. The third originates in the dorsolateral prefrontal cortex, appears to be reciprocally linked to emotional networks and may consequently underlie cognitive and executive symptoms of depression.^57^ Taken together with the above results, it appears that thalamic hyperactivity is associated with treatment resistance in patients with TRD and non-TRD. Underlying this are the emotional salience network, emotion modulation network and cognitive/executive network, in which the thalamus is an integral part of the circuitry.

Regarding the specific pathophysiology of TRD, we showed that patients with TRD had stronger spontaneous neural activity in right triangular part of the IFG, right IPL and vermis compared with patients with non-TRD and healthy participants. Two-sample *t*-tests (TRD compared with non-TRD and TRD compared with HC groups) revealed that patients with TRD had increased resting-state spontaneous neural activity in the right triangular part of the IFG and vermis than non-TRD and HC subjects. Previous studies have shown right frontal gyrus activity impairment in depression.^58, 59^ Consistent with mood disorders, IFG is thought to contribute to emotion regulation.^60^ The right IFG is more strongly involved in ‘suppression', which can decrease negative affect significantly but prolong neural activity in the amygdala and insula.^61^ Thus, hyperactivity of the right IFG in patients with TRD may indicate an altered state of emotion regulation, which may cause a greater need to voluntarily regulate emotion in the resting-state, or more use of a ‘suppression' strategy to decrease negative affect. Furthermore, the right IFG has a crucial role in localized attention and suppression of responses to new stimuli,^62, 63^ suppression of memory and emotion^64, 65^ and comprehension of affective prosody.^66, 67, 68, 69^ Considering these reports, our data suggest that patients with TRD are likely to have changes in attention, emotion, sensitivity to new stimuli and prosody comprehension during resting states.

The IPL is a brain region involved in the DMN,^70, 71^ which consists of areas that are active when an individual is awake and alert, but not actively involved in an attention demanding or goal-directed task, and which deactivate during the performance of cognitive tasks.^72^ The DMN is detectable using task-free functional connectivity MRI and has been implicated in self-referential activity, episodic memory retrieval and emotion modulation.^71, 72^ Altered DMN activity is thought to be a result of rumination in MDD.^73^ Thus, one interpretation of our results is that patients with TRD are highly self-referential and exhibit a ruminative state during wakeful rest. Furthermore, previous research suggests that the supramarginal gyrus is associated with phonological working memory rather than visual working memory.^74^ These combined results predict that patients with TRD may have altered phonological and prosodic processing reflected by right IFG and supramarginal gyrus spontaneous neural activity. The results of this study are also consistent with previous reports suggesting that patients with MDD and a long history of antidepressant use have increased anterior vermis volume compared with HC subjects.^75^ Although the anterior vermis is thought to have a role in sensorimotor functions,^76, 77^ recent research also suggests that this region may mediate non-motor functions such as verbal learning and memory,^78^ social cognition,^79^ reward systems^80^ and addiction.^80^ Thus, our findings suggest that treatment resistance may be associated with alterations in the vermis, which affects both motor and cognitive functions.

As predicted by previous studies, two-sample *t*-tests revealed that patients with MDD (both TRD and non-TRD) had stronger spontaneous precuneus and right IPL (angular gyrus and supramarginal gyrus) neural activity than HC subjects. Precuneus and IPL are associated with the DMN,^70, 71^ which is involved in self-referential activity, episodic memory retrieval and emotion modulation.^71^ Previous research has demonstrated that patients with MDD have hyperactivity in the DMN.^71, 73^ A recent meta-analysis also concluded that hyper-connectivity in DMN brain regions is characteristic of patients with MDD,^43^ potentially as a result of excessive rumination.^73^ The common increased spontaneous neural activity identified in patients with MDD (TRD and non-TRD) in our study is consistent with previous reports. Thus, upregulation of DMN spontaneous neural activity may be a common characteristic of both MDD and TRD, reflecting this highly ruminative state during wakeful rest.

We also identified an additional set of brain regions with altered spontaneous neural activity in TRD. Patients with TRD showed increased fALFF scores in the occipital and calcarine cortices compared with non-TRD and HC groups. This finding is consistent with previous research reporting that fALFF values in this region can accurately distinguish patients with TRD from healthy control subjects (sensitivity: 81.8% specificity: 73.3%).^44^ The recovery rate following 8 weeks of fluoxetine administration was found to be associated with occipital cortex gray matter volume.^30^ Thus, our findings provide further evidence that the occipital and calcarine cortices are associated with TRD.

Among patients with non-TRD, we identified decreased fALFF values in left postcentral gyrus. Previous research reported that patients with MDD show decreased KCC-ReHo values in this region.^19^ Interestingly, patients with TRD showed decreased voxel-mirrored homotopic connectivity in the postcentral gyrus.^81^ Thus, our study provides further evidence of alterations in this brain region. Because KCC-ReHo scores are similar for neural activity time series of particular brain areas and neighboring regions,^17^ this result may reflect activity instability in the postcentral gyrus.

Several limitations should be considered when interpreting the present study results. First, on the basis of small sample size, we cannot conclude definitively that the results of this study reflect the trait marker of TRD. Second, we used a cross-sectional design, making the distinction between identifying treatment-resistance vulnerability markers and progressive occurrences during the course of the illness difficult. However, by adding a prospective follow-up of the non-TRD group, we provide indirect evidence that higher resting-state spontaneous neural activity in the thalamus may be a valid vulnerability marker for treatment resistance, rather than an artifact occurring during the course of TRD. Third, medication use may be a potential confounding variable, as patients with TRD had differing antidepressant regimens during fMRI scans. An appropriately designed study, which is prospectively designed, medication-controlled and uses a larger sample size is therefore needed to test the reliability of these results and determine the trait marker. Fourth, because of our interest in the DMN, we did not use ALFF values in this study. However, in the right lingual gyrus, patients with TRD showed lower spontaneous neural activity compared with non-TRD, and lower activity correlated with lower percent change in HRSD_17_ scores in patients with non-TRD (see Supplementary Table 3). This result supports a previous study^82^ that demonstrated patients with treatment-nonresponsive MDD increase spontaneous neural activity in the right lingual gyrus compared with patients having treatment-responsive MDD. There was also a positive correlation between decreased HRSD_17_ scores and mean ALFF of the right lingual gyrus in patients with treatment-nonresponsive MDD.^82^ To reveal the function of low-frequency oscillations, further studies should use both measures. Fifth, although patients in this study were recruited according to Thase and Rush criteria, additional research is required to reveal the effect of severity of symptoms according to other criteria, as used elsewhere.^16, 20^ Finally, although we used thresholds recommended for balancing type I and type II error,^47^ our findings must be considered preliminary because we did not use multiple comparisons. We propose the following future lines of inquiry: first, comprehensive research coupling resting-state neural activity, task-related activation and network analysis would be needed to reveal alterations of emotion regulation in patients with TRD. Second, to prevent treatment resistance, it is necessary to develop an accurate diagnostic classification system for treatment response, such as machine learning using multiple clinical variables encompassing different modalities.

In summary, this study reveals that spontaneous thalamic hyperactivity has a key role not only in patients with TRD, but also in the early phase of antidepressant treatment resistance among patients with MDD. In addition, we show that spontaneous activity in the right IFG, IPL and vermis may contribute to the specific neural substrates underlying TRD. These results suggest altered cognitive and emotion regulation neural circuits in patients with TRD. Regarding clinical relevance, the results of this study might contribute to personalized treatment in patients with MDD, based on neurobiological features. Resting-state fMRI is much easier to acquire in a routine clinical setting than standard fMRI paradigms.^83^ Thus, resting-state fMRI along with fALFF could be used as complementary assessment methods for predicting treatment response in the early phase of treatment. Further, fALFF may allow the suggestion of suitable treatments based on the neurological profile of individual patients.

## Figures and Tables

**Table 1:** Demographic information for the three comparison groups

	*HC*	*Non-TRD*	*Non-TRD (6w)*	*TRD*	P*-value*
*N* (male/female)	26 (11/15)	16 (7/9)		16 (10/6)	0.41^a^
Age (mean (*s.d.*))	45.3 (10.2)	45.7 (11.7)		44.6 (9.7)	0.95^b^
HRSD_17_ score (mean (*s.d.*))		15.4 (3.1)	10.6 (4.8)	13.6 (3.8)	0.23^c^
Age of onset (mean (*s.d.*))		42.3 (13.1)		39.3 (9.9)	0.47^c^
Duration of current episode (median (*s.d.*))		3.0 (16.0)		58.5 (10.8)	<0.01^d^
JART score (mean (*s.d.*))	112.2 (9.4)	119.2 (5.1)		110.8 (10.1)	0.02^b^

Abbreviations: 6w, 6-week follow-up; HC, healthy control; HRSD, Hamilton Rating Scale for Depression; JART, Japanese Adult Reading Test; TRD, treatment-resistant depression.

a Pearson's chi-square test for HC, non-TRD and TRD groups.

b One-way analysis of variance for HC, non-TRD and TRD groups.

c Two-sample *t*-test for non-TRD and TRD groups.

d Mann–Whitney *U*-test for non-TRD and TRD groups.

**Table 2:** Clinical characteristics of patients with TRD

*Parameter/dimension*	n	*%*	*Score*
*Duration of episode*			
Acute (≤12 months)	0	0.0	1
Subacute (13–24 months)	2	12.5	2
Chronic (≥24 months)	14	87.5	3
			
*Symptom severity*^a^			
Subsyndromal	1	6.2	1
Mild	2	12.5	2
Moderate	6	37.5	3
Severe without psychosis	7	43.8	4
Severe with psychosis	0	0.0	5
			
*Antidepressant medication use*			
Level 1: 1–2 medications	4	25.0	1
Level 2: 3–4 medications	6	37.5	2
Level 3: 5–6 medications	4	25.0	3
Level 4: 7–10 medications	2	12.5	4
Level 5: >10 medications	0	0.0	5
			
*Augmentation*			
Used	11	68.8	0
Not used	5	31.2	1
			
*Electroconvulsive therapy*			
Used	1	6.2	0
Not used	15	93.8	1
			
*Model summary*^b^ *(mean=8.50, s.d.=2.48)*			
Mild resistance (scores=3–6)	5	31.2	
Moderately resistance (scores=7–10)	9	56.3	
Severe resistance (scores=11–15)	2	12.5	

Abbreviation: TRD, treatment-resistant depression.

a Symptom severity was categorized into five groups to fit the severity classes identified *a priori* according to the Mental and Behavioral Disorders section of the 10th revision of the International Classification of Diseases. The subsyndromal subtype was a residual group including patients who were symptomatic but did not fulfill the diagnostic criteria for any of the other diagnostic subtypes.^40^

b Model summary score was sum of the duration, symptom severity, antidepressant medication use, augmentation and electroconvulsive therapy scores.^40^

**Table 3:** Brain regions showing differences in fALFF values among groups

*Contrasts for group comparisons and identified brain* *regions labeled by AAL*^a^	*Direction*	*MNI coordinates*^b^ *(cluster maxima)*			*Cluster size (mm*^*3*^)	t *(cluster maxima)*	P*-value*
		x	y	z			
*TRD>non-TRD*							
Inferior frontal gyrus, triangular part	R	54	30	0	945	4.19	<0.001
Middle occipital gyrus	R	−30	−90	24	297	4.03	<0.001
Thalamus	R	21	−18	12	270	3.98	<0.001
Supramarginal gyrus	R	54	−45	27	324	3.73	<0.001
Vermis/lingual/cerebellum	L/R	0	−45	27	675	3.70	<0.001
							
*TRD>HC*							
Inferior frontal gyrus, triangular part	R	57	30	3	891	4.69	<0.001
Middle occipital gyrus/angular gyrus/inferior parietal lobule	R	48	−78	27	4509	4.62	<0.001
Vermis/cerebellum	L/R	3	−51	6	2673	4.39	<0.001
Precuneus	L/R	0	−57	48	5049	4.11	<0.001
Thalamus	R	21	−21	3	270	4.04	<0.001
Cerebellum	L	−9	−42	−33	459	4.01	<0.001
							
*HC>TRD*							
Precentral	L	−42	−18	63	324	4.79	<0.001
Calcarine cortex/inferior occipital gyrus	R	15	−102	−3	1512	4.45	<0.001
Calcarine cortex	L	−9	−99	−6	945	4.44	<0.001
Paracentral lobule	L	−15	−27	63	1107	4.15	<0.001
Pre/postcentral gyrus	L	−30	−24	42	1026	3.97	<0.001

*Non-TRD>HC*							
Middle occipital gyrus	L	−33	−63	27	486	4.22	<0.001
Precuneus/cuneus	L	−6	−63	48	3024	3.88	<0.001
Precuneus	L	−9	−60	15	540	3.67	<0.001
Angular gyrus	R	42	−69	33	783	3.01	0.002
							
*HC>non-TRD*							
Pre/postcentral gyrus	L	−36	−15	45	783	3.96	<0.001
Precentral gyrus	L	−15	−27	57	297	3.52	<0.001

Abbreviations: AAL, anatomical automatic labeling; fALFF, fractional amplitude of low-frequency fluctuations; HC, healthy control; L, left; MNI, Montreal Neurological Institute, R, right; TRD, treatment-resistant depression.

a *P*_uncorrected_<0.005, k≥10.

b Coordinates (*x*, *y* and *z*) show primary peak voxel locations of each cluster in the MNI space.
