# Supplementary material for: Association of thalamic hyperactivity with treatment-resistant depression and poor response in early treatment for major depression: a resting-state fMRI study using fractional amplitude of low-frequency fluctuations
Source: Transl Psychiatry. 2016 Mar 8;6(3):e754–. doi: 10.1038/tp.2016.18 (PMC4872444; doi:10.1038/tp.2016.18)
Supplement: Supplementary Table 1 [file tp201618x1.doc]

**Supplementary Table 1.**

**Patient medication use.**

| Medication category | Individual medications (*n*) |
| --- | --- |
| **non-TRD group** |  |
| Antidepressants | SSRI (16) |
| Benzodiazepines | Benzodiazepines (11) |
|  |  |
| **TRD group** |  |
| Antidepressants | TCA (4) |
|  | SSRI (11) |
|  | SNRI (3) |
|  | NaSSA (4) |
|  | Others (4) |
| Mood stabilizers | Lithium (2) |
|  | Sodium valproate (2) |
|  | Topiramate (1) |
| Antipsychotics | Antipsychotics (9) |
| Stimulants | Methylphenidate (1) |
| Benzodiazepines | Benzodiazepines (14) |
| Abbreviations: NaSSA, noradrenergic and specific serotonergic antidepressant; SNRI, serotonin and noradrenalin reuptake inhibitor; SSRI, serotonin reuptake inhibitor; TCA, tricyclic antidepressants. | |
