# Supplementary material for: Association of thalamic hyperactivity with treatment-resistant depression and poor response in early treatment for major depression: a resting-state fMRI study using fractional amplitude of low-frequency fluctuations
Source: Transl Psychiatry. 2016 Mar 8;6(3):e754–. doi: 10.1038/tp.2016.18 (PMC4872444; doi:10.1038/tp.2016.18)
Supplement: Supplementary Table 2 [file tp201618x2.doc]

**Supplementary Table 2.**

**Brain regions showing differences in ALFF values among groups.**

| Contrasts for group comparisons and identified brain regions labeled by AAL^1^ | Direction | MNI coordinates^2^ (cluster maxima) | | | Cluster size (mm^3^) | *t* (cluster maxima) | *P*-value |
| --- | --- | --- | --- | --- | --- | --- | --- |
|  |  | x | y | z |  |  |  |
| **non-TRD > TRD** |  |  |  |  |  |  |  |
| Lingual | R | 12 | −87 | −12 | 297 | 3.50 | < 0.001 |
| Postcentral | L | −39 | −30 | 60 | 567 | 3.36 | 0.001 |
| **TRD > HC** |  |  |  |  |  |  |  |
| ParaHippocampal / olfactory | R | 18 | 9 | −30 | 1296 | 4.33 | < 0.001 |
| Insula | R | 42 | −9 | −6 | 486 | 3.98 | < 0.001 |
| Insula | R | 39 | 6 | −3 | 324 | 3.41 | 0.001 |
| **HC > TRD** |  |  |  |  |  |  |  |
| Precentral / precentral lobule | L | −39 | −21 | 60 | 7371 | 4.87 | < 0.001 |
| Precentral | R | 33 | −24 | 51 | 3375 | 4.33 | < 0.001 |
| Inferior parietal lobule | R | 24 | −51 | 51 | 594 | 4.74 | < 0.001 |
| Precentral | R | 51 | −12 | 42 | 1728 | 3.95 | < 0.001 |
| Lingual | R | 12 | −87 | −12 | 297 | 3.51 | < 0.001 |
| **non-TRD > HC** |  |  |  |  |  |  |  |
| Parahippocampal | R | 24 | −27 | −21 | 729 | 4.76 | < 0.001 |
| Fusiform / parahippocampal | L | −21 | −30 | −24 | 972 | 4.37 | < 0.001 |
| Insula | R | 39 | 9 | −3 | 621 | 4.34 | < 0.001 |
| **HC > non-TRD** |  |  |  |  |  |  |  |
| Precentral | R | 36 | −18 | 36 | 1890 | 5.03 | < 0.001 |
| Postcentral / paracentral lobule | L | −36 | −21 | 39 | 3780 | 4.08 | < 0.001 |
| Postcentral | R | 18 | −30 | 57 | 2268 | 3.95 | < 0.001 |
| Abbreviations: AAL, anatomical automatic labeling; MNI, Montreal Neurological Institute.  ^1^*P_uncorrected_* < 0.005, k ≥ 10  ^2^Coordinates (x, y, and z) show primary peak voxel locations of each cluster in the MNI space. | | | | | | | |
