# Supplementary material for: Association of thalamic hyperactivity with treatment-resistant depression and poor response in early treatment for major depression: a resting-state fMRI study using fractional amplitude of low-frequency fluctuations
Source: Transl Psychiatry. 2016 Mar 8;6(3):e754–. doi: 10.1038/tp.2016.18 (PMC4872444; doi:10.1038/tp.2016.18)
Supplement: Supplementary Table 3 [file tp201618x3.doc]

**Supplementary Table 3.**

**Correlation between ALFF and percent change in HRSD_17_ scores in the non-TRD group.**

| Contrasts for group comparisons and identified brain regions labeled by AAL^1^ | Partial correlation coefficient  *r*^2^ | *P-*value  for *r* |
| --- | --- | --- |
| **non-TRD > TRD** |  |  |
| Lingual | 0.583 | 0.001 |
| Postcentral | 0.144 | 0.560 |
| Abbreviations: AAL, anatomical automatic labeling.  ^1^*P_uncorrected_* < 0.005, k ≥ 10.  ^2^Removing the effect of duration of current episode (month). | | |
